# Arbuscular Mycorrhizal Symbiosis Alleviates Salt Stress in Black Locust through Improved Photosynthesis, Water Status, and K^+^/Na^+^ Homeostasis

**DOI:** 10.3389/fpls.2017.01739

**Published:** 2017-10-10

**Authors:** Jie Chen, Haoqiang Zhang, Xinlu Zhang, Ming Tang

**Affiliations:** ^1^State Key Laboratory of Soil Erosion and Dryland Farming on the Loess Plateau, Northwest A&F University, Yangling, China; ^2^College of Forestry, Northwest A&F University, Yangling, China

**Keywords:** arbuscular mycorrhizal fungi, black locust, photosynthesis, water status, ion homeostasis, salt stress

## Abstract

Soil salinization and the associated land degradation are major and growing ecological problems. Excess salt in soil impedes plant photosynthetic processes and root uptake of water and nutrients such as K^+^. Arbuscular mycorrhizal (AM) fungi can mitigate salt stress in host plants. Although, numerous studies demonstrate that photosynthesis and water status are improved by mycorrhizae, the molecular mechanisms involved have received little research attention. In the present study, we analyzed the effects of AM symbiosis and salt stress on photosynthesis, water status, concentrations of Na^+^ and K^+^, and the expression of several genes associated with photosynthesis (*RppsbA, RppsbD, RprbcL*, and *RprbcS*) and genes coding for aquaporins or membrane transport proteins involved in K^+^ and/or Na^+^ uptake, translocation, or compartmentalization homeostasis (*RpSOS1, RpHKT1, RpNHX1*, and *RpSKOR*) in black locust. The results showed that salinity reduced the net photosynthetic rate, stomatal conductance, and relative water content in both non-mycorrhizal (NM) and AM plants; the reductions of these three parameters were less in AM plants compared with NM plants. Under saline conditions, AM fungi significantly improved the net photosynthetic rate, quantum efficiency of photosystem II photochemistry, and K^+^ content in plants, but evidently reduced the Na^+^ content. AM plants also displayed a significant increase in the relative water content and an evident decrease in the shoot/root ratio of Na^+^ in the presence of 200 mM NaCl compared with NM plants. Additionally, mycorrhizal colonization upregulated the expression of three chloroplast genes (*RppsbA, RppsbD*, and *RprbcL*) in leaves, and three genes (*RpSOS1, RpHKT1*, and *RpSKOR*) encoding membrane transport proteins involved in K^+^/Na^+^ homeostasis in roots. Expression of several aquaporin genes was regulated by AM symbiosis in both leaves and roots depending on soil salinity. This study suggests that the beneficial effects of AM symbiosis on the photosynthetic capacity, water status, and K^+^/Na^+^ homeostasis lead to the improved growth performance and salt tolerance of black locust exposed to salt stress.

## Introduction

Soil salinization and the associated land degradation are major and growing ecological problems, particularly in arid and semiarid areas (Porcel et al., 2012, 2015). Excess salt in the soil impedes plant growth and development (Evelin et al., 2009) by causing a series of physiological stresses in plants (Aroca et al., 2013). Photosynthesis, the key process of primary metabolism (Pinheiro and Chaves, 2011), is affected by salinity in several ways (Chaves et al., 2009). First, CO_2_ availability declines because of the limitations of diffusion through the stomata and mesophyll (Chaves et al., 2009), and second, salt stress hinders the photosynthetic electron flow through photosystems II and I (PSII and PSI) (Lu and Vonshak, 2002; Allakhverdiev and Murata, 2008). Moreover, the D1 and D2 proteins, which are in the PSII reaction center and are pivotal in the phosphorylation-coupled linear electron flow (Jansen et al., 1996), degrade under salt stress (Sudhir et al., 2005; Neelam and Subramanyam, 2013).

Another challenge faced by plants exposed to salt stress is acquiring sufficient amounts of water from the soil (Ouziad et al., 2006). Root uptake of water is limited by a diminished water potential due to the accumulation of salt in the soil (Ruiz-Lozano et al., 2012). Aquaporins are proposed to play a pivotal role in plant water equilibrium and water use efficiency (WUE) (Sade et al., 2010). The passive transport of water molecules across membranes is facilitated by these intrinsic membrane proteins (Maurel et al., 2008). Plant aquaporins are categorized into five subfamilies (Ruiz-Lozano et al., 2012), of which plasma membrane intrinsic proteins (PIPs) and tonoplast intrinsic proteins (TIPs) are the most abundant in plant plasma and vacuolar membranes, respectively (Luu and Maurel, 2005), and serve as the primary path for transcellular and intracellular water movement, respectively (Maurel et al., 2008).

Plants exposed to salt stress also suffer from Na^+^ toxicity and K^+^ deficiency, since the acquisition of K^+^ is disrupted by excess Na^+^ in the soil (Porcel et al., 2016). Over-accumulation of Na^+^ in plants damages cellular organelles, impairs photosynthesis, and competes with K^+^ for protein biosynthesis and enzyme activation (Shabala and Cuin, 2008; Porcel et al., 2016). The regulation of several genes encoding membrane transport proteins responsible for K^+^ and/or Na^+^ uptake, translocation or compartmentalization is included among the strategies of plants to deal with excessive accumulation of Na^+^ and a deficit in K^+^. Plasma membrane Na^+^/H^+^ antiporter SOS1 and tonoplast (Na^+^, K^+^)/H^+^ antiporter NHX1 are responsible for efflux of Na^+^ from cells and sequestration of this cation in the vacuole, respectively (Qiu et al., 2002; Deinlein et al., 2014). They are two major mechanisms that maintain low Na^+^ accumulation in the cytosol (Deinlein et al., 2014). Moreover, class I HKT transporters are involved in removing Na^+^ from the xylem in order to prevent Na^+^ from transport to the leaves (Sunarpi et al., 2005; Davenport et al., 2007), while the outward-rectifying K^+^ channel SKOR controls K^+^ loading into the xylem and K^+^ transport to shoots (Zhang et al., 2017).

Arbuscular mycorrhizal (AM) fungi symbiotically associate with most terrestrial plants (Ruiz-Lozano et al., 2012). AM symbiosis not only improves the water and nutrient uptake (Smith and Read, 2008) but also mitigates environmental stresses (e.g., salinity) in the host plants (Porcel et al., 2012). Previous studies have demonstrated that mycorrhizal symbiosis can increase photosynthesis in plants exposed to salt stress (Sheng et al., 2008; Hajiboland et al., 2010; Porcel et al., 2015). However, the molecular mechanisms involved remain to be determined. First, the regulation of *psbA* (encoding D1 protein) and *psbD* (encoding D2 protein) by AM fungi can be expected because of a frequently observed increase in the quantum yield of PSII photochemistry from AM symbiosis (Sheng et al., 2008; Hajiboland et al., 2010; Porcel et al., 2015). Additionally, Porcel et al. (2015) found that the expression of *rbcL* (encoding the large subunit of ribulose-1,5-bisphosphate carboxylase/oxygenase, rubisco) and *rbcS* (encoding the small subunit of rubisco) was not upregulated by mycorrhizal colonization in the leaves of rice. Therefore, we conducted an additional investigation to explore the influence of AM fungi on gene expression when plants are subjected to salt stress.

Arbuscular mycorrhizal symbiosis also improves the water uptake and water status in plants exposed to salt stress (Aroca et al., 2007; Sheng et al., 2008; Wu et al., 2015), and such improvement is likely related to changes in the expression of aquaporin genes (Ruiz-Lozano et al., 2012). Under salt stress, the regulation of plant aquaporin genes by AM symbiosis has been observed (Ouziad et al., 2006; Aroca et al., 2007; Jahromi et al., 2008). However, most of these studies focused on the regulation of aquaporin genes in roots, and the regulation in leaves has rarely been studied. The regulation of aquaporin genes in leaves may mediate membrane permeability to water and CO_2_ and further affect cellular water conservation and the mesophyll conductance of CO_2_ diffusion (Jang et al., 2004; Saibo et al., 2009; Yang and Cui, 2009). Thus, the regulation of aquaporin genes in leaves must be considered in the further exploration of the effects of AM symbiosis on plant water status and photosynthesis. Additionally, the response of aquaporin genes in roots to AM symbiosis and salt stress is inconsistent. For example, mycorrhizal colonization downregulates *LePIP1* under salt stress (Ouziad et al., 2006), whereas mycorrhizae upregulates *LsPIP1* under saline conditions (Jahromi et al., 2008). Such contradictions may be a consequence of the different plant and fungi species used and the different methods of salt application (Ouziad et al., 2006). However, most studies investigated AM symbiosis and the regulation of aquaporins in response to salinity using agronomic plant species. The regulation of these genes by AM fungi in response to salinity is unknown in a tree species.

Moreover, mycorrhizal symbiosis can facilitate K^+^ uptake while preventing Na^+^ absorption and translocation to the shoots (Evelin et al., 2012; Garcia et al., 2017). Exploring the regulation of genes encoding membrane transport proteins involved in K^+^/Na^+^ homeostasis in AM plants subjected to salinity is an essential step in understanding the mechanisms underlying salt tolerance conferred by AM fungi, but such research is lacking. Porcel et al. (2016) found that the expression of *OsSOS1, OsNHX3, OsHKT2;1*, and *OsHKT1;5* was upregulated by mycorrhizal colonization in plants subjected to salt stress, concomitantly with the reduced roots to shoots distribution of Na^+^ in AM plants. Estrada et al. (2013) found that AM fungi native to saline areas were better able to regulate root *ZmAKT2, ZmSKOR*, and *ZmSOS1* expression levels than non-native AM fungi, as demonstrated by stronger correlation with higher K^+^/Na^+^ ratios in plants inoculated with native AM fungi than in plants inoculated with non-native AM fungi and non-mycorrhizal (NM) plants. Ouziad et al. (2006) found that the expression of *LeNHX1* and *LeNHX2* was not altered by AM symbiosis.

Black locust (*Robinia pseudoacacia* L.), a woody legume, has been widely planted as a pioneer tree in arid and semiarid regions in China (Tian et al., 2003). The species is fast growing, is tolerant to drought and salt stress and can improve soil through N-fixation in root nodules (Taniguchi et al., 2007; Jin et al., 2011; Gu et al., 2012). Black locust also has great economic value because the leaves, flowers, and wood can be used to feed livestock, to produce honey and as lumber, respectively (Yang et al., 2014; He et al., 2016). AM fungi can establish a mutualistic symbiosis with black locust and can alleviate drought stress or metal toxicity (He et al., 2017; Huang et al., 2017); however, little is known about the influence of the mutualism under salt stress, particularly the molecular mechanisms involved.

Therefore, the purposes of the present study were the following: (1) to determine the effects of AM symbiosis on photosynthesis, gas-exchange parameters, PSII operating efficiency and the expression pattern of four genes (*RppsbA, RppsbD, RprbcL*, and *RprbcS*) associated with photosynthesis in the leaves of black locust subjected to salt stress, (2) to examine the responses of six aquaporin genes to AM symbiosis and salinity in the leaves and roots of black locust, and (3) to determine the regulation of K^+^ and Na^+^ content and the K^+^/Na^+^ ratio, as well as the expression of four genes (*RpSOS1, RpHKT1, RpNHX1*, and *RpSKOR*) encoding transport proteins involved in K^+^ and/or Na^+^ uptake, translocation or compartmentalization by AM symbiosis in the leaves and roots of black locust exposed to salt stress.

## Materials and Methods

### Experimental Design

A pot experiment was conducted at Northwest A&F University using a randomized complete block design with three salinity levels (0, 100, and 200 mM NaCl) and two inoculation status (NM and mycorrhizal plants) for six treatments total, with 30 replicates per treatment (one plant per pot for a total of 180 pots).

### Growth Substrate

The growth substrate was soil (<2 mm) mixed with fine sand (<2 mm) in the proportion of 1:1 (v/v). The soil/sand mixture was autoclaved (0.11 MPa, 121°C) for 2 h. The original soil collected from Northwest A&F University had a pH of 7.6 (1:5, soil:water, w/v) and contained 34.9 mg kg^-1^ available nitrogen, 15.8 mg kg^-1^ available phosphorous, 165.7 mg kg^-1^ available potassium and 17.5 g kg^-1^ organic matter.

### Plant Material

Seeds of *R*. *pseudoacacia* acquired from Northwest A&F University (Yangling, China) were surface sterilized in a 5% NaClO solution for 10 min, washed three times with sterile water, pre-germinated on sterilized moist filter paper in petri dishes and transplanted into pots (10 cm × 12 cm, three germinated seeds per pot) containing 630 g of the growth substrate. After 1 week, the plants were thinned to one seedling per pot.

### Inoculation Treatment

The AM inoculum, *Rhizophagus irregularis* (BGC BJ109), was obtained from the Beijing Academy of Agriculture and Forestry Sciences (Beijing, China) and was multiplied in pot cultures of *Zea mays* L. (Yang et al., 2014). The inoculum contained sand, spores (approximately 50 spores per gram), mycelia, and colonized root fragments. Each AM plant received 8 g of the inoculum at the time of the transplant, whereas each NM plant received the same amount of the autoclaved inoculum and the filtrate (<20 μm) of the AM inoculum to obtain a native microbial population free of mycorrhizal propagules.

### Growth Conditions

The experiment was conducted in a greenhouse with a relative humidity of 50-75% and temperatures ranging from 20 to 35°C. The plants were watered daily and fertilized every 3 weeks with 50 ml per pot of Hoagland solution containing 1/2 phosphate throughout the experimental period. Plants were grown for 10 weeks before salinization treatment. Then, three levels (0, 100, and 200 mM NaCl) of saline solution were added to the corresponding pots for 7 days (15 ml/day; a total of 105 ml was added). The electrical conductivity values were 1.12, 5.63, and 8.95 dS m^-1^ for the growth substrate treated with 0, 100, and 200 mM NaCl, respectively. After an additional 3 weeks of growth, the plants were harvested.

### Parameters Measured

#### Mycorrhizal Colonization

Fresh roots, used to determine mycorrhizal colonization (five plants per treatment, *n* = 5), were gently washed with distilled water, cut into 1-cm pieces, cleared in 5% KOH, then acidified with 1% HCl and stained in 0.05% trypan blue in lactophenol following the method of Phillips and Hayman (1970) before observation under a microscope. The gridline intersect method (Giovannetti and Mosse, 1980) was used to determine the mycorrhizal colonization.

#### Plant Biomass and Leaf Water Status

At harvest, the leaves, stems and roots of plants (five plants per treatment, *n* = 5) were separated, and the fresh weights (FWs) were measured. Then, the leaves were soaked in distilled water for 24 h for the turgid weight (TW) determination. Finally, the three plant parts were oven-dried at 70°C to a constant weight to measure the dry weights (DWs). The shoot dry mass was calculated as the sum of the DWs of leaves and stems. The relative water content (RWC) and water saturation deficit (WSD) were calculated using the following formulas (Gao, 2006):

$$\begin{array}{c} \text{RWC}(\%)\text{=}\frac{\text{FW-DW}}{\text{TW-DW}}\times \text{100}\\ \text{WSD}(\%)\text{=}\frac{\text{TW-FW}}{\text{TW-DW}}\times \text{100}. \end{array}$$

#### Photosynthesis and Gas Exchange Parameters

The net photosynthetic rate (Pn), stomatal conductance (Gs), intercellular CO_2_ concentration (Ci), and transpiration rate (Tr) were measured from 8:30 to 11:30 a.m. with a Li-6400 portable open flow gas-exchange system (Li-Cor Inc., Lincoln, NE, United States) before harvest. The fifth or sixth youngest leaf of each plant (five plants per treatment, *n* = 5) was used for the measurements. The measurement conditions were as follows: photosynthetically active irradiation, 1000 μmol m^-2^ s^-1^; temperature, 25°C; relative humidity, 60%; and CO_2_ concentration, 400 μmol mol^-1^. The WUE was calculated as the ratio of Pn to Tr (Sheng et al., 2008).

#### Chlorophyll Fluorescence Parameters

Chlorophyll fluorescence parameters were measured with a modulated chlorophyll fluorimeter (Mini-Imaging-PAM, Walz, Germany). The fifth or sixth youngest leaf of each plant (four plants per treatment, *n* = 4) was used for the measurements. Plants were adapted to the dark for 30 min before the measurement of F_0_ and Fm. The maximum quantum efficiency of PSII photochemistry (Fv/Fm), PSII operating efficiency (ΦPSII), PSII maximum efficiency (Fv′/Fm′), photochemical quenching coefficient (qP), and non-photochemical quenching (NPQ) were calculated with F_0_, Fm and the light-adapted parameters using the following formulas (Baker, 2008):

$$\begin{array}{c} \frac{\text{Fv}}{\text{Fm}}=(\text{Fm}-{\text{F}}_{0})/\text{Fm}\\ \Phi \text{PSII=}({\text{Fm}}^{\prime }-{\text{F}}^{\prime }){\text{/Fm}}^{\prime }\\ \frac{{\text{Fv}}^{\prime }}{{\text{Fm}}^{\prime }}=({\text{Fm}}^{\prime }-{\text{F}}_{\text{0}}{}^{\prime })/{\text{Fm}}^{\prime }\\ \begin{array}{c} \text{qP}=\frac{{\text{Fq}}^{\prime }}{{\text{Fv}}^{\prime }}=({\text{Fm}}^{\prime }-{\text{F}}^{\prime })/({\text{Fm}}^{\prime }-{\text{F}}_{\text{0}}{}^{\prime })\\ \text{NPQ}=\frac{\text{Fm}}{{\text{Fm}}^{\prime }}-1 \end{array} \end{array}$$

#### Concentration of Na^+^ and K^+^

Oven-dried leaves and roots (five plants per treatment, *n* = 5) were ground to powder and samples (0.1 g) were acid-digested with 8 ml HNO_3_ + 2 ml HClO_4_, and heated to 220°C for extraction of Na^+^ and K^+^ ions. After cooling to room temperature, extraction solution was diluted before determination of the Na^+^ and K^+^ content with a Z-2000 atomic absorption spectrophotometer (Shimadzu, Japan).

#### RNA Extraction and cDNA Synthesis

At harvest, the leaves and roots of plants (three plants per treatment, *n* = 3) were frozen in liquid nitrogen and stored at -80°C until use. After the frozen leaves and roots were ground to a fine powder in liquid nitrogen, RNA was extracted with an E.Z.N.A^TM^ plant RNA kit (Omega Bio-Tek, Norcross, GA, United States). RNA integrity and purity were checked by 1% agarose gel electrophoresis and spectrophotometric analysis using a NanoDrop 2000 (Thermo Scientific, Pittsburgh, PA, United States). Reverse transcription to complementary DNA (cDNA) was conducted using a PrimeScript^TM^ RT reagent kit with gDNA eraser (TaKaRa Bio, Dalian, China).

#### Cloning of Partial Coding Sequences (CDSs) of *RprbcS, RpSOS1, RpHKT1*, and *RpSKOR*

After ClustalW alignment of published CDSs for *rbcS* (*Ammopiptanthus mongolicus, Arachis hypogaea, Phaseolus vulgaris, Vigna radiata, Pisum sativum, Cicer arietinum, Medicago truncatula, Glycine max*, and *Medicago sativa*), *SOS1* (*Artemisia japonica, Glycine max, Vigna angularis*, and *Vigna radiate*), *HKT1* (*Arachis ipaensis, Arachis duranensis, Lupinus angustifolius*, and *Medicago truncatula*), and *SKOR* (*Arachis ipaensis, Arachis duranensis, Cicer arietinum, Lupinus angustifolius, Vigna angularis*, and *Vigna radiata*) (all from NCBI^1^) using MEGA 5.0 (Tamura et al., 2011), four pairs of degenerate primers (Supplementary Table S1) were designed based on nucleotide sequence conservation. Then, the degenerate primers were used to amplify the cDNA in a gradient PCR reaction (20 μl) containing 10 μl of Premix Taq^®^Version 2.0, 0.4 μM of each primer, 1.6 μl of a 1:5 dilution of cDNA and 6.8 μl of ddH_2_O. The PCR reaction was conducted using a C1000 thermocycler (Bio-Rad Laboratories, Hercules, CA, United States) with the following procedure: a 2 min denaturation at 95°C, followed by 30 cycles of denaturation at 95°C for 30 s, annealing at 40 to 60°C for 30 s and extension at 72°C for 30 s, followed by a final extension at 72°C for 10 min. PCR products were inserted into the pEASY-T1 cloning vector (TransGen Biotech, Beijing, China) and transformed into Trans5α chemically competent cells (TransGen Biotech, Beijing, China). Then, LB agar plates containing 15 mg ml^-1^ agarose, 10 mg ml^-1^ NaCl, 10 mg ml^-1^ peptone, 5 mg ml^-1^ yeast extract and 100 μg ml^-1^ ampicillin were used to select the transformants. Beijing AuGCT DNA-SYN Biotechnology Co., Ltd. (Beijing, China) sequenced the clones with inserts, and the obtained sequences were blasted using blastx on NCBI^2^. Putative partial CDSs of *RprbcS* (MF948148), *RpSOS1* (MF948149), *RpHKT1* (MF948150), and *RpSKOR* (MF948151) have been deposited in GenBank.

#### Gene Expression

The primers used in the qRT-PCR were designed with Primer Premier 5.0 (Premier Biosoft International, Palo Alto, CA, United States) and are listed in Supplementary Table S2. The qRT-PCR reaction was conducted using the CFX96 real-time PCR detection system (Bio-Rad Laboratories) and contained 10 μl of SYBR^®^Premix Ex Taq^TM^ II (TakaRa Bio, Dalian, China), 0.4 μM of each primer, 1.6 μl of a 1:5 dilution of cDNA and 6.8 μl of ddH_2_O. The PCR procedure consisted of a 30 s denaturation at 95°C, followed by 40 cycles of denaturation at 95°C for 5 s and annealing/elongation at the annealing temperature (Supplementary Table S2) for 60 s. For *RprbcS*, the PCR procedure was switched to a 30 s denaturation at 95°C, followed by 40 cycles of 5 s at 95°C, 30 s at 57°C and 60 s at 72°C. A heat melt curve (from 65 to 95°C) was used to check the specificity of the PCR amplification. All samples were technically replicated three times. Negative controls without cDNA were run within each analysis. Normalization was performed based on the expression of the *R*. *pseudoacacia* actin gene (Meng et al., 2016). The relative quantity of transcripts was determined using the 2^-ΔΔC_T_^ method (Livak and Schmittgen, 2001).

### Statistical Analyses

The SPSS 17.0 statistical software (SPSS Inc., Chicago, IL, United States) was used for statistical analyses. Data were subjected to a one-way ANOVA and *post hoc* comparisons (Duncan’s test, *P* < 0.05), in addition to a two-way ANOVA with three sources of variation (salt, AM fungi inoculation and their interaction). Figures were drawn with SigmaPlot 10.0 (Systat Software Inc., San Jose, CA, United States) and the package “pheatmap” in R.

## Results

### Mycorrhizal Colonization and Plant Biomass

**FIGURE 1:**
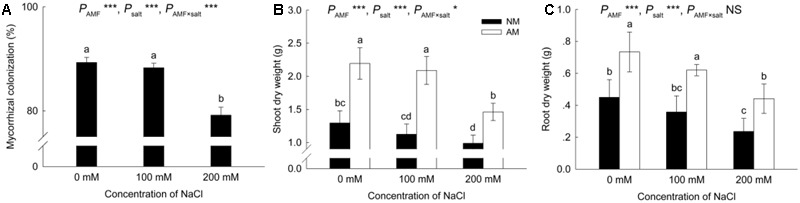
Effects of salinity and *Rhizophagus irregularis* inoculation on mycorrhizal colonization **(A)** and shoot **(B)** and root **(C)** dry mass in *Robinia pseudoacacia*. Different letters indicate significant differences (*P* < 0.05). NS not significant; ^∗^*P* < 0.05; ^∗∗^*P* < 0.01; ^∗∗∗^*P* < 0.001. AM, arbuscular mycorrhizal; NM, non-mycorrhizal.

Arbuscular mycorrhizal plants showed 89.29% mycorrhizal colonization under non-saline conditions, which decreased to 79.16% under the 200 mM NaCl treatment (**Figure 1A**). No significant differences in colonization rate were detected between the 0 and 100 mM NaCl treatments. No mycorrhizal colonization was found in NM plants.

Increased salinity decreased the shoot and root dry mass in both NM and AM plants (**Figures 1B,C**). Nevertheless, the shoot and root DWs of AM plants were greater than those of NM plants at all salinity levels.

### Photosynthesis and Gas-Exchange Parameters

**FIGURE 2:**
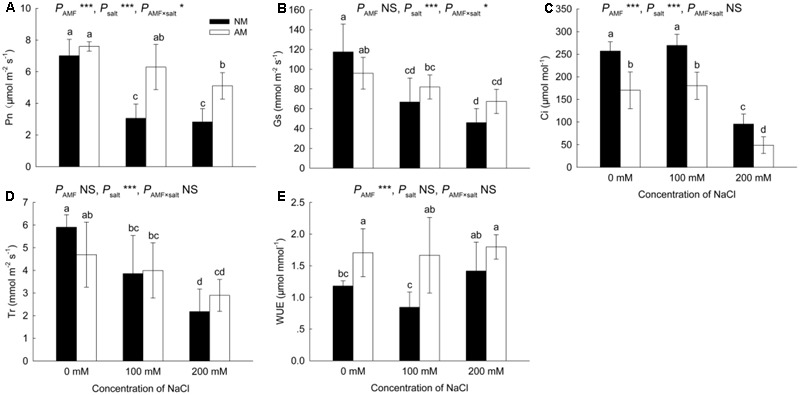
Effects of salinity and *R*. *irregularis* inoculation on net photosynthetic rate (Pn) **(A)**, stomatal conductance (Gs) **(B)**, intercellular CO_2_ concentration (Ci) **(C)**, transpiration rate (Tr) **(D)**, and water use efficiency (WUE) **(E)** in leaves of *R*. *pseudoacacia*. Different letters indicate significant differences (*P* < 0.05). NS not significant; ^∗^*P* < 0.05; ^∗∗^*P* < 0.01; ^∗∗∗^*P* < 0.001. AM, arbuscular mycorrhizal; NM, non-mycorrhizal.

Salinity negatively affected the Pn in NM and AM plants, causing reductions in the Pn of NM plants of 56% and 60% at 100 and 200 mM NaCl, respectively (**Figure 2A**). However, in AM plants, the reductions due to salinity were only significant (33%) at 200 mM NaCl. AM symbiosis improved the Pn by 106% at 100 mM NaCl and by 81% at 200 mM NaCl.

Salinity reduced the Gs and Tr in NM and AM plants (**Figures 2B,D**). Similar to the results for the Pn, salinity caused a greater reduction in the Gs and Tr in NM plants than in AM plants. However, the differences in the Tr and Gs were not significant between NM and AM plants.

The application of 200 mM NaCl reduced the Ci in NM and AM plants (**Figure 2C**), although the Ci was higher in NM plants than in AM plants at all salinity levels.

The increases in salinity had little effect on the WUE (**Figure 2E**). However, the WUE of AM plants was higher than that of NM plants at 0 and 100 mM NaCl.

### Chlorophyll Fluorescence Parameters

**FIGURE 3:**
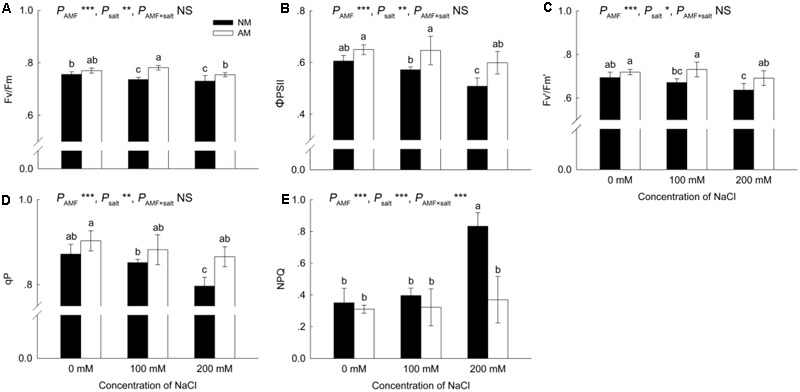
Effects of salinity and *R*. *irregularis* inoculation on maximum quantum efficiency of PSII photochemistry (Fv/Fm) **(A)**, PSII operating efficiency (ΦPSII) **(B)**, PSII maximum efficiency (Fv′/Fm′) **(C)**, photochemical quenching coefficient (qP) **(D)** and non-photochemical quenching (NPQ) **(E)** in leaves of *R*. *pseudoacacia*. Different letters indicate significant differences (*P* < 0.05). NS not significant; ^∗^*P* < 0.05; ^∗∗^*P* < 0.01; ^∗∗∗^*P* < 0.001. AM, arbuscular mycorrhizal; NM, non-mycorrhizal.

Increased salinity reduced the Fv/Fm, ΦPSII, Fv′/Fm′ and qP in NM plants; however, in AM plants, increased salinity did not cause a significant decrease in these four parameters (**Figures 3A–D**). Values of Fv/Fm, ΦPSII and Fv′/Fm′ were higher in AM plants than in NM plants under saline conditions, and qP was increased by AM symbiosis at 200 mM NaCl.

The NPQ was similar in NM and AM plants under 0 and 100 mM NaCl, but at 200 mM NaCl, the NPQ increased in NM plants and was higher (125%) than that in AM plants (**Figure 3E**).

### Leaf Water Status

**FIGURE 4:**
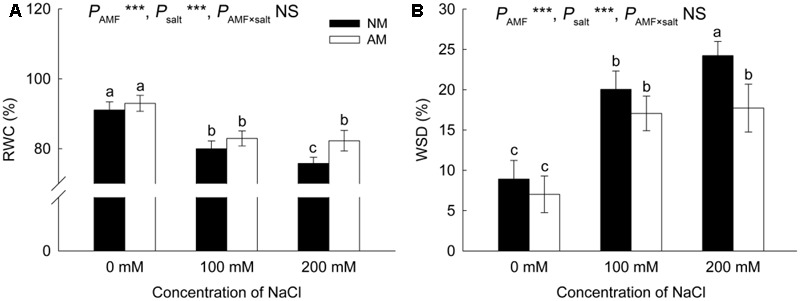
Effects of salinity and *R*. *irregularis* inoculation on relative water content (RWC) **(A)** and water saturation deficit (WSD) **(B)** in leaves of *R*. *pseudoacacia*. Different letters indicate significant differences (*P* < 0.05). NS not significant; ^∗^*P* < 0.05; ^∗∗^*P* < 0.01; ^∗∗∗^*P* < 0.001. AM, arbuscular mycorrhizal; NM, non-mycorrhizal.

The RWC declined with exposure to salinity (**Figure 4A**). In NM plants, the reduction in the RWC ranged from 12% at 100 mM NaCl to 17% at 200 mM NaCl, whereas in AM plants, the reduction was 11 and 12% under 100 and 200 mM NaCl, respectively. AM fungi led to a 9% increase in the RWC at 200 mM NaCl.

The WSD increased when plants were exposed to salt stress (**Figure 4B**). Under the 200 mM NaCl treatment, the WSD was lower (27%) in AM plants than in NM plants.

### Concentration of Na^+^ and K^+^, K^+^/Na^+^ Ratio, and Shoot/Root Na^+^ Ratio

**FIGURE 5:**
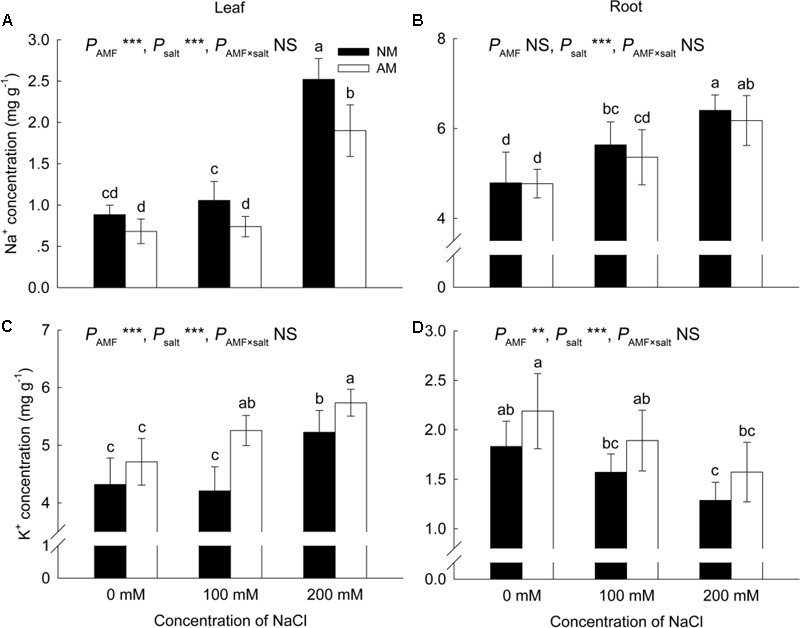
Effects of salinity and *R*. *irregularis* inoculation on concentration of Na^+^ **(A,B)** and K^+^ **(C,D)** in leaves and roots of *R*. *pseudoacacia*. Different letters indicate significant differences (*P* < 0.05). NS not significant; ^∗^*P* < 0.05; ^∗∗^*P* < 0.01; ^∗∗∗^*P* < 0.001. AM, arbuscular mycorrhizal; NM, non-mycorrhizal.

Salinity increased the Na^+^ concentration in leaves and roots of both NM and AM plants (**Figures 5A,B**). Under non-saline conditions, AM plants accumulated a similar amount of Na^+^ in leaves to NM plants, however, under saline conditions, AM plants accumulated less Na^+^ in leaves than NM plants. Meanwhile, in roots, Na^+^ accumulation was similar in NM and AM plants at all salinity levels.

Upon increasing salinity, the concentration of K^+^ decreased in the roots of both NM and AM plants, but increased in the leaves of both plants (**Figures 5C,D**). In leaves, K^+^ accumulation was similar in NM and AM plants under non-saline conditions, but under saline conditions, the K^+^ concentration in AM plants was higher than that in NM plants. Meanwhile in roots, AM plants accumulated a similar amount of K^+^ as NM plants at all salinity levels.

**FIGURE 6:**
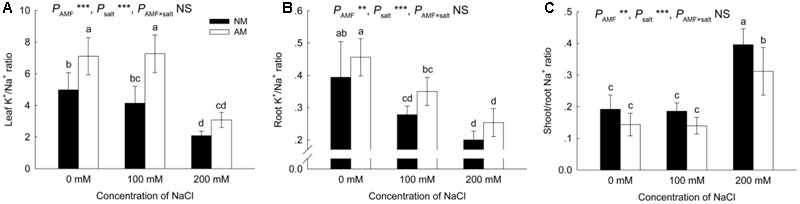
Effects of salinity and *R*. *irregularis* inoculation on leaf **(A)** and root **(B)** K^+^/Na^+^ ratio and shoot/root Na^+^ ratio **(C)** in *R*. *pseudoacacia*. Different letters indicate significant differences (*P* < 0.05). NS not significant; ^∗^*P* < 0.05; ^∗∗^*P* < 0.01; ^∗∗∗^*P* < 0.001. AM, arbuscular mycorrhizal; NM, non-mycorrhizal.

Salinity negatively affected the K^+^/Na^+^ ratio in the leaves and roots of NM and AM plants (**Figures 6A,B**). In roots, the K^+^/Na^+^ ratio was similar in NM and AM plants at all salinity levels, while in leaves, the ratio was improved by AM symbiosis at 0 and 100 mM NaCl.

The shoot/root Na^+^ ratio increased upon exposure to salinity (**Figure 6C**), and AM symbiosis reduced the shoot/root Na^+^ ratio at 200 mM NaCl.

### Expression of *RppsbA, RppsbD, RprbcL*, and *RprbcS*

**FIGURE 7:**
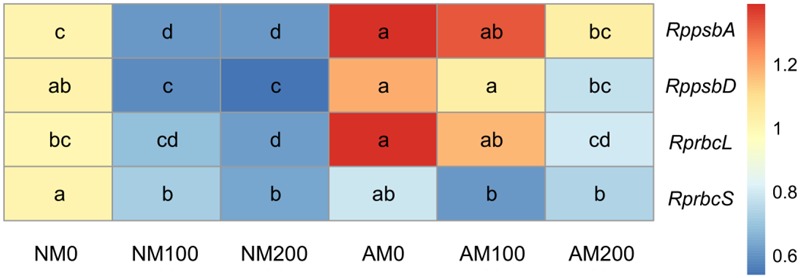
Effects of salinity and *R*. *irregularis* inoculation on expression of *RppsbA, RppsbD, RprbcL*, and *RprbcS* in leaves of *R*. *pseudoacacia*. Expression of the *R*. *pseudoacacia* actin gene was used as an internal control for normalization. Different letters within each gene indicate significant differences (*P* < 0.05). AM, arbuscular mycorrhizal; NM, non-mycorrhizal.

The expression of *RppsbA* and *RppsbD* was downregulated in NM and AM plants in the salinity treatments (**Figure 7**). AM fungi upregulated the expression of *RppsbA* under non-saline and saline conditions, and the expression of *RppsbD* was upregulated by mycorrhizae at 100 mM NaCl.

Salinity also downregulated the expression of *RprbcL* and *RprbcS* in NM plants, but only reduced the expression of *RprbcL* in AM plants (**Figure 7**). The expression level of *RprbcL* was higher in AM plants than in NM plants at 0 and 100 mM NaCl, whereas the expression of *RprbcS* was similar in NM and AM plants at all salinity levels.

### Expression of Aquaporin Genes

**FIGURE 8:**
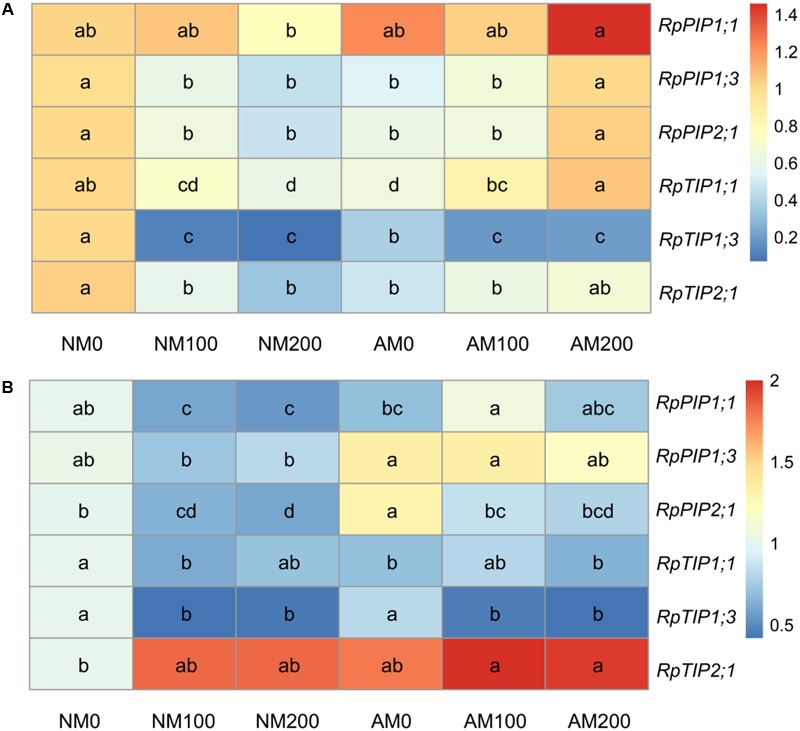
Effects of salinity and *R*. *irregularis* inoculation on expression of aquaporin genes in roots **(A)** and leaves **(B)** of *R*. *pseudoacacia*. Expression of the *R*. *pseudoacacia* actin gene was used as an internal control for normalization. Different letters within each gene indicate significant differences (*P* < 0.05). AM, arbuscular mycorrhizal; NM, non-mycorrhizal.

In the roots of NM plants, almost all aquaporin genes tested (except for *RpPIP1;1*, which was unaffected by salinity) were downregulated in response to salinity (**Figure 8A**), whereas in the roots of AM plants, the expression was reduced less (*RpTIP1;3*), was unaffected (*RpPIP1;1* and *RpTIP2;1*) or was even increased (*RpPIP1;3, RpPIP2;1*, and *RpTIP1;1*) with increasing salinity. Under non-saline conditions, mycorrhizal fungi caused a downregulation of almost all aquaporin genes (except for *RpPIP1;1*) in roots. However, at 200 mM NaCl, the expression of four aquaporins (*RpPIP1;1, RpPIP1;3, RpPIP2;1*, and *RpTIP1;1*) was higher in AM plants than in NM plants.

In the leaves of NM plants, the expression of *RpPIP1;1, RpPIP2;1, RpTIP1;1*, and *RpTIP1;3* was downregulated by the application of 100 or 200 mM NaCl, while in the leaves of AM plants, salinity only reduced the expression of *RpPIP2;1* and *RpTIP1;3* (**Figure 8B**). The opposite trend was observed in the leaf expression of *RpPIP1;1*, which was upregulated by the application of 100 mM NaCl in AM plants. Under non-saline conditions, mycorrhizal fungi increased the leaf expression of *RpPIP2;1*, whereas the expression of *RpTIP1;1* decreased in response to mycorrhizae. Under saline conditions, the upregulation in leaf expression of *RpPIP1;1* and *RpPIP1;3* by mycorrhizae was observed at 100 mM NaCl. No significant differences in the leaf expression of *RpTIP1;3* or *RpPIP2;1* were found between NM and AM plants.

### Expression of *RpSOS1, RpHKT1, RpNHX1*, and *RpSKOR*

**FIGURE 9:**
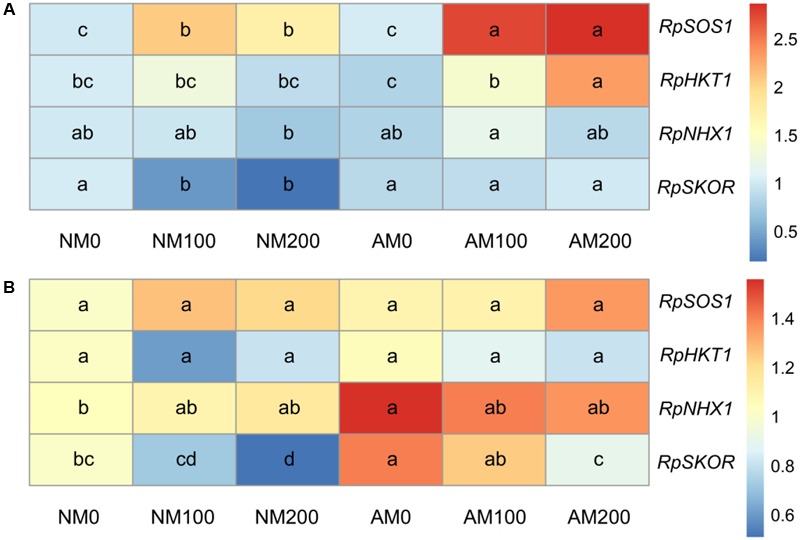
Effects of salinity and *R*. *irregularis* inoculation on expression of *RpSOS1, RpHKT1, RpNHX1*, and *RpSKOR* in roots **(A)** and leaves **(B)** of *R*. *pseudoacacia*. Expression of the *R*. *pseudoacacia* actin gene was used as an internal control for normalization. Different letters within each gene indicate significant differences (*P* < 0.05). AM, arbuscular mycorrhizal; NM, non-mycorrhizal.

In roots, salinity upregulated the expression of *RpSOS1* and *RpHKT1* in AM plants but only increased the expression of *RpSOS1* in NM plants (**Figure 9A**). Salinity decreased the expression of *RpSKOR* in the roots of NM plants, but had no effect on the expression this gene in the roots of AM plants. Mycorrhizal colonization upregulated the expression of *RpSOS1* and *RpSKOR* under saline conditions, and increased the expression of *RpHKT1* at 200 mM NaCl. Neither salinity nor AM symbiosis had a significant effect on the expression of *RpNHX1* in roots.

In leaves, salinity downregulated the expression *RpSKOR* in NM and AM plants, but did not affect the expression of *RpSOS1, RpHKT1*, or *RpNHX1* in both plants (**Figure 9B**). AM symbiosis upregulated the expression of *RpSKOR* at all salinity levels, and enhanced the expression of *RpNHX1* under non-saline conditions. No significant differences in the leaf expression of *RpSOS1* or *RpHKT1* were found between NM and AM plants.

## Discussion

Salinity decreases the colonization capacity of AM fungi by suppressing hyphal growth, sporulation, and spore germination (Juniper and Abbott, 2006; Jahromi et al., 2008). In the present research, salinity caused a reduction in mycorrhizal colonization, consistent with previous studies on lettuce and *Trigonella foenum-graecum* (Aroca et al., 2013; Evelin and Kapoor, 2014).

Plant biomass production is the most obvious trait reflecting plant performance under abiotic stress and the symbiosis efficiency of AM fungi (Evelin et al., 2009; Ruiz-Lozano et al., 2012; Porcel et al., 2016; Zhang et al., 2017). In the present study, although the dry masses of the shoots and roots of both NM and AM plants were reduced by salt stress, AM plants grew better than NM plants at all salinity levels, which suggests that the AM symbiosis mitigated salt stress in black locust and indicates a high symbiosis efficiency of *R*. *irregularis*. Beneficial influence of AM symbiosis on biomass production in plants exposed to salt stress are also found in other plant species, including tomato, rice, lettuce, and maize (Hajiboland et al., 2010; Sheng et al., 2011; Aroca et al., 2013; Porcel et al., 2016).

The reduction in plant growth under saline conditions is attributed to a reduction in photosynthetic capacity caused by salinity (Porcel et al., 2015). In the present study, although salinity reduced the Pn in both NM and AM plants, the Pn of AM plants was higher than that of NM plants under saline conditions, which correlated with the improved biomass production in the shoots and roots of AM plants exposed to saline conditions. Salt stress affects CO_2_ diffusion in leaves through a decline in stomatal and mesophyll conductance (Chaves et al., 2009), and the Pn enhancement by mycorrhizal fungi is likely related to a higher Gs in AM plants than in NM plants (Hajiboland et al., 2010; Porcel et al., 2015). However, Wu et al. (2015) showed that under saline conditions, mycorrhizal fungi improved the Pn in male *Populus cathayana* but did not significantly improve the Gs in host plants, although the Gs decreased less in AM plants compared with that in NM plants when salinity increased (Wu et al., 2015). Consistent with the results of Wu et al. (2015), we also observed less reduction in the Gs in AM plants than in NM plants with increasing levels of salinity and similar Gs values in AM and NM plants under saline conditions. The lower reduction in Gs in AM plants than in NM plants with increasing salinity suggests CO_2_ diffusion through the stomata was diminished less, hence the water status may be better in AM plants. Moreover, in this study, AM plants had a lower Ci than NM plants under all salinity levels, which is consistent with a previous study of AM-positive effects on plant photosynthesis by Sheng et al. (2008). Under saline conditions, a high Ci may indicate the destruction of the photosynthetic apparatus and passivation of the enzymes, which could both decrease CO_2_ assimilation and induce CO_2_ accumulation in intercellular areas (Sheng et al., 2008). In the present study, *RprbcL* expression was upregulated in AM plants at 0 and 100 mM NaCl. The *RprbcL* gene encodes the large subunit of rubisco, which catalyzes the first step of photosynthetic carbon assimilation in C_3_ plants (Sudhir and Murthy, 2004), and its upregulation may contribute to improved CO_2_ assimilation. Thus, the lower Ci in AM plants might be a consequence of a lower metabolic limitation of CO_2_ assimilation in these plants compared with the NM plants.

Photochemical reactions reflect the photosynthetic capacity and energy use efficiency in plants and are sensitive to abiotic stresses (Hu et al., 2017). A correlation between maintenance of PSII activity and adaptation to abiotic stresses has been reported in previous studies (Sheng et al., 2008; Hajiboland et al., 2010; Porcel et al., 2016; Hu et al., 2017). For the chlorophyll fluorescence parameters measured in this study, AM symbiosis had a positive effect on the Fv/Fm and ΦPSII under saline conditions, and on the qP in the presence of 200 mM NaCl, which implies that AM plants had a less stressed PSII, a higher quantum efficiency of PSII photochemistry and a greater ability to drive electron transport in an excited PSII reaction center under the stress conditions (Baker, 2008). Moreover, the NPQ increased in NM plants with increasing salinity but was unaffected in AM plants. Normally, an increase in the NPQ is a photoprotective mechanism used to dissipate the excess excitation energy as heat, but this increase can lead to a decrease in the quantum efficiency of PSII photochemistry (Baker, 2008). Therefore, AM symbiosis increased the use of absorbed light in photochemical processes with minimal dissipation of light energy (Hu et al., 2017). In addition, mycorrhizal colonization upregulated the expression of *RppsbA* at all salinity levels, and increased the expression of *RppsbD* at 100 mM NaCl. This upregulation by AM symbiosis may make AM plants more capable of repairing PSII following degradation of D1 and D2 proteins by salinity. Thus, our results suggest that plants inoculated with AM fungi were more tolerant to salt stress due to a higher PSII efficiency under saline conditions, which increased photosynthetic capacity in these plants.

The beneficial effects of AM symbiosis on plant growth and photosynthesis can also be ascribed to an improvement in water status with mycorrhizal colonization. A positive effect on the RWC due to AM symbiosis in the 200 mM NaCl treatment was observed in this study. Moreover, the lower reduction in RWC in AM plants exposed to salinity correlated with the lower reduction in Gs in these plants. Given that aquaporins mediate root hydraulic conductance and plant water homeostasis (Luu and Maurel, 2005; Sade et al., 2010), mycorrhizal regulation of aquaporin genes could be expected.

Plasma membrane intrinsic proteins are expressed in abundance in roots and regulate most of the water absorption by roots, whereas TIPs regulate water exchange between the vacuole and cytosol and are important in cellular osmoregulation (Luu and Maurel, 2005). Downregulation of PIPs and TIPs under salt stress may be a mechanism to reduce membrane permeability for water and to conserve cellular water (Jang et al., 2004; He et al., 2016). In the present study, we also observed decreased expression of aquaporin genes in both the leaves and roots of NM plants, as well as *RpPIP2;1* (in leaves) and *RpTIP1;3* (in leaves and roots) in AM plants under salt stress. However, salinity also resulted in the upregulation of one TIP (*RpTIP1;1* at 100 and 200 mM NaCl) and two PIPs (*RpPIP1;3* and *RpPIP2;1* at 200 mM NaCl) in AM roots, and one PIP (*RpPIP1;1* at 100 mM NaCl) in AM leaves. The upregulation of PIPs under saline conditions likely assists in water uptake to sustain water homeostasis in cells with high salinity levels (Jang et al., 2004), and the upregulation of TIPs under such conditions may facilitate water transport from the cytosol to the vacuole to increase osmotic pressure (Hu et al., 2017). Variable responses by different members of the aquaporins to salt or drought stress have been observed in previous studies (Jang et al., 2004; Ouziad et al., 2006; Bárzana et al., 2014; He et al., 2016), which may reflect the complexity of the transcriptional regulation of different members of the large aquaporin family (Ouziad et al., 2006).

In the present study, AM fungi regulated several aquaporin genes in both leaves and roots. The root expression of *RpPIP1;1, RpPIP1;3, RpPIP2;1*, and *RpTIP1;1* was upregulated by AM symbiosis at 200 mM NaCl. In previous studies, under short-term drought (Bárzana et al., 2014) and salt stress (Aroca et al., 2007), an increase in the root expression of PIPs and TIPs by mycorrhizae was correlated with higher free-exuded sap flow rate and osmotic root hydraulic conductance in AM plants than in NM plants. Therefore, the upregulation of aquaporin genes by mycorrhizal fungi at 200 mM NaCl may reflect the stress conditions under which AM roots take up more water than NM roots, which would contribute to the improved water status of AM plants compared with NM plants. In the present study, mycorrhizal fungi increased the leaf expression of *RpPIP1;1* and *RpPIP1;3* at 100 mM NaCl. Aquaporins can transport CO_2_ and are involved in CO_2_ diffusion across the plasma membrane of mesophyll cells (Saibo et al., 2009; Yang and Cui, 2009). In plants overexpressing *NtAQP1*, the membrane permeability for CO_2_ diffusion was increased (Uehlein et al., 2003), while the presence of HgCl_2_, which inhibits most aquaporins, reduced the permeability (Terashima and Ono, 2002). Therefore, the upregulation of aquaporin genes by mycorrhizal fungi in leaves suggests that AM plants have a better supply of water from the roots (Ouziad et al., 2006) and a higher mesophyll permeability for CO_2_ diffusion, which would reduce CO_2_ accumulation in intercellular areas and contribute to a higher Pn in these plants compared with NM plants.

Salt tolerance in plants depends to a great extent on the absorption and distribution of Na^+^ within the plant (Porcel et al., 2016). Previous studies reported a reduction in Na^+^ accumulation (Talaat and Shawky, 2011; Garcia et al., 2017) and reduced root-to-shoot allocation of Na^+^ (Evelin et al., 2012; Porcel et al., 2016) in AM plants. We also found that AM plants accumulated less Na^+^ in leaves compared with NM plants under salt conditions, concomitantly with a reduced shoot/root Na^+^ ratio in AM plants at 200 mM NaCl. Preventing Na^+^ transport from roots to shoots is a strategy for protecting photosynthetic organs against the toxic effects of Na^+^ (Zhu et al., 2016). Ion selection can be controlled by AM fungi during nutrient absorption from the soil (Hammer et al., 2010). In the present study, leaf K^+^ levels were significantly higher in AM plants than in NM plants under saline conditions, and the leaf K^+^/Na^+^ ratio was improved by AM symbiosis at 0 and 100 mM NaCl. Increased accumulation of K^+^ and reduced Na^+^ concentration in AM plants have also been reported in other studies (Evelin et al., 2012; Garcia et al., 2017). K^+^ is a macronutrient that is essential for many plant processes including osmoregulation, protein biosynthesis, enzyme activation, and photosynthesis (Shabala and Cuin, 2008; Wang and Wu, 2013). Na^+^ competes with K^+^ for metabolic processes requiring K^+^ (Shabala and Cuin, 2008), but Na^+^ cannot replace the function of K^+^ (Estrada et al., 2013). Thus, maintenance of a higher concentration of K^+^ and a higher K^+^/Na^+^ ratio in AM plants exposed to salinity was presumably crucial for salt tolerance.

Based on the better performance of AM plants in reducing the shoot/root Na^+^ ratio and in improving the K^+^/Na^+^ ratio in leaves, we decided to investigate the response of four genes involved in K^+^ and/or Na^+^ uptake, translocation, or compartmentalization. The results showed that the root expression of *RpSOS1* was upregulated under increased salinity in both NM and AM plants, and the expression was also enhanced by AM symbiosis under saline conditions. Upregulation of *RpSOS1* in roots promotes Na^+^ export back to the soil or to the apoplastic spaces (Shi et al., 2002). As a consequence, AM plants may be more capable of reducing Na^+^ influx into roots and/or restricting the toxicity of Na^+^, since Na^+^ is less toxic in the apoplastic spaces. In the present study, we also observed that mycorrhizal colonization increased the root expression of *RpHKT1* at 200 mM NaCl, which correlated with the lower shoot/root Na^+^ ratio in AM plants at 200 mM NaCl and suggests that unloading of Na^+^ from the xylem was enhanced in AM plants in order to prevent allocation of the cation to the shoots (Deinlein et al., 2014). The upregulation of *RpSOS1* and *RpHKT1* in roots by AM symbiosis contributed to reduced Na^+^ accumulation in the leaves of AM plants. The results of the present study also showed that the root expression of *RpSKOR* was increased by AM symbiosis under saline conditions. This upregulation increased K^+^ loading into the xylem (Zhang et al., 2017) and correlated with improved K^+^ accumulation in the leaves of AM plants observed in the present study. In addition, the enhanced expression of *RpSKOR* in combination with *RpSOS1* and *RpHKT1* in AM plants can account for the higher K^+^/Na^+^ ratio in the leaves of these plants. Curiously, AM symbiosis had on effect on the expression of *RpNHX1* in leaves or roots under saline conditions, consistent with research on tomato by Ouziad et al. (2006). However, Porcel et al. (2016) found that AM symbiosis considerably upregulated *OsNHX3* expression in leaves under saline conditions. Indeed, in rice, tonoplast-localized OsNHX1 - 4 function as vacuolar (Na^+^, K^+^)/H^+^ antiporters (Porcel et al., 2016). Therefore, the regulation of additional genes encoding for NHX proteins in *R*. *pseudoacacia* by mycorrhizal fungi requires further exploration in order to illustrate their contribution to Na^+^ compartmentalization in AM plants. Interestingly, it seems that mycorrhizal colonization had a more evident influence on the expression of *RpSOS1* and *RpHKT1* in roots than in leaves, since no significant induction by AM symbiosis was detected in leaves. This is inconsistent with the study on rice plants by Porcel et al. (2016) in which the expression of genes involved in Na^+^ uptake or translocation in leaves was influenced by AM symbiosis to a greater extent than that in roots, and this apparent contradiction may be a consequence of the different plant and fungi species used in the experiments.

## Conclusion

Arbuscular mycorrhizal fungi improved salt tolerance of black locust by increasing photosynthetic capacity, water status and K^+^/Na^+^ homeostasis, as well as reducing the root-to-shoot allocation of Na^+^. The improvement in photosynthesis by AM fungi was related to a lower reduction in Gs, a higher PSII efficiency and higher expression of three chloroplastic genes (*RprbcL, RppsbA*, and *RppsbD*) compared with NM plants. Mycorrhizal fungi regulated the expression of several aquaporin genes, depending on the salinity level applied and the plant tissue. Under saline conditions, the upregulation of aquaporin genes by AM symbiosis in leaves and roots might facilitate water uptake by roots, water transport to special tissues and CO_2_ diffusion across the plasma membrane. AM symbiosis also facilitated Na^+^ secretion from root cells, Na^+^ unloading from the xylem, and K^+^ translocation to the shoots. This is associated with the upregulation of *RpSOS1, RpHKT1*, and *RpSKOR* expression in roots by AM fungi under stress conditions. The beneficial effects of AM symbiosis on the photosynthetic capacity, water status and ionic homeostasis resulted in the improved growth performance of black locust exposed to salt stress.

## Author Contributions

MT conceived the study. JC conducted the experiments, analyzed the data and drafted manuscript. XZ participated in the experiment. HZ and MT reviewed and edited the manuscript. All authors read and approved the manuscript for final submission.

## Conflict of Interest Statement

The authors declare that the research was conducted in the absence of any commercial or financial relationships that could be construed as a potential conflict of interest.

## Footnotes

1 https://www.ncbi.nlm.nih.gov

2 https://blast.ncbi.nlm.nih.gov/Blast.cgi

## Abbreviations

AM: arbuscular mycorrhizal
Ci: intercellular CO_2_ concentration
DW: dry mass
Fv/Fm: maximum quantum efficiency of PSII photochemistry
Fv′/Fm′: PSII maximum efficiency
FW: fresh weight
Gs: stomatal conductance
NM: non-mycorrhizal
NPQ: non-photochemical quenching
Pn: net photosynthetic rate
qP: photochemical quenching coefficient
RWC: relative water content
Tr: transpiration rate
TW: turgid weight
WSD: water saturation deficit
WUE: water use efficiency
ΦPSII: PSII operating efficiency
