# Supplementary material for: Arbuscular Mycorrhizal Symbiosis Alleviates Salt Stress in Black Locust through Improved Photosynthesis, Water Status, and K+/Na+ Homeostasis
Source: Front Plant Sci. 2017 Oct 10;8:1739. doi: 10.3389/fpls.2017.01739 (PMC5641402; doi:10.3389/fpls.2017.01739)
Supplement: Supplementary file 1 [file Table_1.docx]

**Supplemental Materials**

**Table S1** Sequences of primers used for cloning of partial coding sequences of *RprbcS*, *RpSOS1*, *RpHKT1* and *RpSKOR*.

| Primer name | Primer sequence (5′-3′) |
| --- | --- |
| *RprbcS* For | CWVTTGGVARGAAGAAGTT |
| *RprbcS* Rev | ATGAARCTRATGCAYTGMACTTG |
| *RpSOS1* For | AAGGTTGGAATYTGSWTGTTA |
| *RpSOS1* Rev | AATWGMRCTTTSCTSCCACAG |
| *RpHKT1* For | GTTTCAACWTTTGCAAGTTGTGG |
| *RpHKT1* Rev | TCTTGAGYCTYCCRAARAACAT |
| *RpSKOR* For | CTATGGAGAYATACATGCAGT |
| *RpSKOR* Rev | AAGCTGCWCTRTTKACCTTCAA |

**Table S2** Sequences and annealing temperatures of primers used in qRT-PCR.

| Primer name | Primer sequence (5′-3′) | Annealing  temperature (°C) |
| --- | --- | --- |
| *RppsbA* For | GTATTCGTGAGCCTGTTTCTGGAT | 55 |
| *RppsbA* Rev | CCGCCATTGTATAACCATTCATC |  |
| *RppsbD* For | ATATGATGGGAGTTGCCGGTGTAT | 56.5 |
| *RppsbD* Rev | TAAGTTTCTTCAGCTTGGGTGGG |  |
| *RprbcL* For | CCAAAGATACTGATATCTTGGCAGCAT | 54 |
| *RprbcL* Rev | GATGTGGTAGCATCGTCCTTTGTA |  |
| *RprbcS* For | TTTCTTACCTGCCACCACTCACC | 57 |
| *RprbcS* Rev | TCCACATAATCCAGTAGCGTCCA |  |
| *RpPIP1;1* For | TTTGAGGGCAATGCTCGGTATG | 59 |
| *RpPIP1;1* Rev | CAGTGGCGGAGAAGACAGTGTAG |  |
| *RpPIP1;3* For | TTCACTTGGCTACCATCCCTATC | 58.5 |
| *RpPIP1;3* Rev | AGGGCATGGCTCTGATTACGACT |  |
| *RpPIP2;1* For | AAGGTGGCACTGAGTGTGATGG | 58.5 |
| *RpPIP2;1* Rev | CCTTGCGTCCAAGGAACAACC |  |
| *RpTIP1;1* For | GGCGGCAACATCACCTTACT | 57 |
| *RpTIP1;1* Rev | AAAGTCCAAATGCGGGAACAG |  |
| *RpTIP1;3* For | CCCTTCTCAGAGGCATTTTGTATTG | 57 |
| *RpTIP1;3* Rev | CTCAAAGACAAGTGCGTTTCCTG |  |
| *RpTIP2;1* For | GCATGTCTCCTCCTCAATTTGGT | 55.4 |
| *RpTIP2;1* Rev | CTGCAGTGGCATAAACAGTGTAAAC |  |
| *RpSOS1* For | GTGCTCTGCCATCTTCTGTTCGTGA | 60 |
| *RpSOS1* Rev | CTTCATAAAGACCCAATGTGCTCCC |  |
| *RpHKT1* For | CTCTTGATTGTCACTGTCTTTGGG | 57 |
| *RpHKT1* Rev | AGTATGTCTGGCGTTTGTAACTTG |  |
| *RpNHX1* For | GCAGCCTTCGTGCCTTACTA | 53 |
| *RpNHX1* Rev | CCATTGTTGACCATTGCGTTC |  |
| *RpSKOR* For | TCATGGTGTGCTGGAGGAAGTAG | 57 |
| *RpSKOR* Rev | ATGATTGTTTATCAAGCCGTAGGAC |  |
| actin For | CCCAAATCATGTTTGAGACCTTCA | 57 |
| actin Rev | CATAGATTGGCACAGTGTGACTCA |  |
